# Identification of diagnostic markers involved in the pathogenesis of gastric cancer through iTRAQ-based quantitative proteomics

**DOI:** 10.1016/j.dib.2016.12.023

**Published:** 2017-01-18

**Authors:** Zhen Jiang, Hongchun Shen, Bo Tang, Hui Chen, Qin Yu, Xingli Ji, Li Wang

**Affiliations:** a Department of Biochemistry, School of Preclinical Medicine, North Sichuan Medical College, Nanchong, Sichuan Province 637100, PR China; b College of Integrated Chinese and Western Medicine, Southwest Medical University, Luzhou, Sichuan Province 646000, PR China; c Department of Pathology, Affiliated Traditional Medicine Hospital, Southwest Medical University, Luzhou, Sichuan 646000, PR China; d Research Center of Combine Traditional Chinese and Western Medicine, Affiliated Traditional Medicine Hospital, Southwest Medical University, Luzhou, Sichuan 646000, PR China

**Keywords:** iTRAQ, isobaric tag for relative and absolute quantitation, LC, liquid chromatography, ESI, electrospray ionization, MS/MS, tandem mass spectrometry, FABP1, fatty acid binding protein, FASN, fatty acid synthase, GC, gastric cancer

## Abstract

We provide detailed datasets from our analysis of proteins that are differentially expressed in gastric cancer tissues compared with adjacent normal gastric tissues, as identified by iTRAQ-based quantitative proteomics. Also included is a set of representative images of immunohistochemical staining of gastric cancer tissues showing four levels of expression of fatty acid binding protein (FABP1) and fatty acid synthase (FASN). The data presented in this paper support the research article “Quantitative proteomic analysis reveals that proteins required for fatty acid metabolism may serve as diagnostic markers for gastric cancer” (Jiang et al., 2017) [1]. We expect that the data will contribute to the identification of sensitive and specific biomarkers for early detection of gastric cancer.

**Specifications Table**

**Table:** 

Subject area	*Biology*
More specific subject area	*Biochemistry and molecular biology*
Type of data	*Tables, figures*
How data was acquired	*Microscope, strong cation exchange (SCX) chromatography, mass spectroscopy*
Data format	*Analyzed*
Experimental factors	*Before LC-ESI-MS/MS, the proteins were labeled with iTRAQ reagents.*
Experimental features	*The authors used iTRAQ labeling combined with LC-ESI-MS/MS analysis to identify differentially expressed proteins in gastric cancer tissues.*
Data source location	*Gastric cancer and adjacent normal tissues were selected from the tissue bank at the Departments of Pathology, Affiliated Traditional Medicine Hospital of the Southwest Medical University, Luzhou, Sichuan 646000, P. R. China.*
Data accessibility	*With this article*

**Value of the data**

• Current biomarkers for gastric cancer show insufficient clinical diagnostic sensitivity and specificity [2]. There is thus an urgent need to identify more reliable clinical biomarkers and to develop accurate and effective approaches to gastric cancer diagnosis, especially for early-stage screening. One promising approach is to identify metabolic proteins dysregulated in cancer cells through quantitative proteomics analysis [3], [4], [5].
• Data from the LC-ESI-MS/MS analysis will provide researchers with detailed information on proteins dysregulated in gastric cancer tissues.
• Data from the immunohistochemical analysis will enable researchers to observe the different levels of FABP1 and FASN staining in gastric cancer tissues.

## 1 Data

**Fig. 1:**
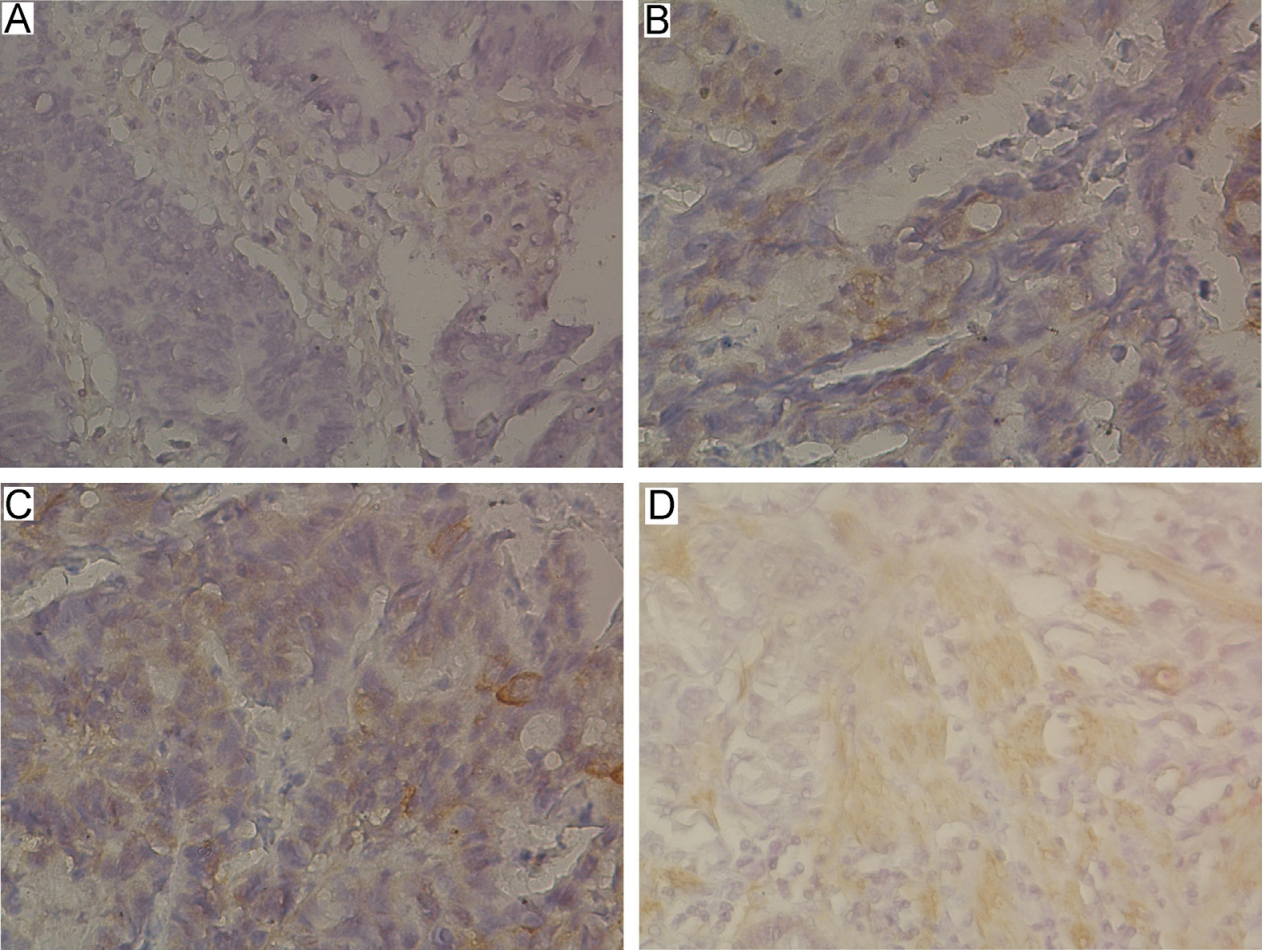
Representative images of immunohistochemical staining levels of FABP1 in gastric cancer tissues. (A) absent [0], (B) weak [1], (C) moderate [2], and (D) strong [3].

**Fig. 2:**
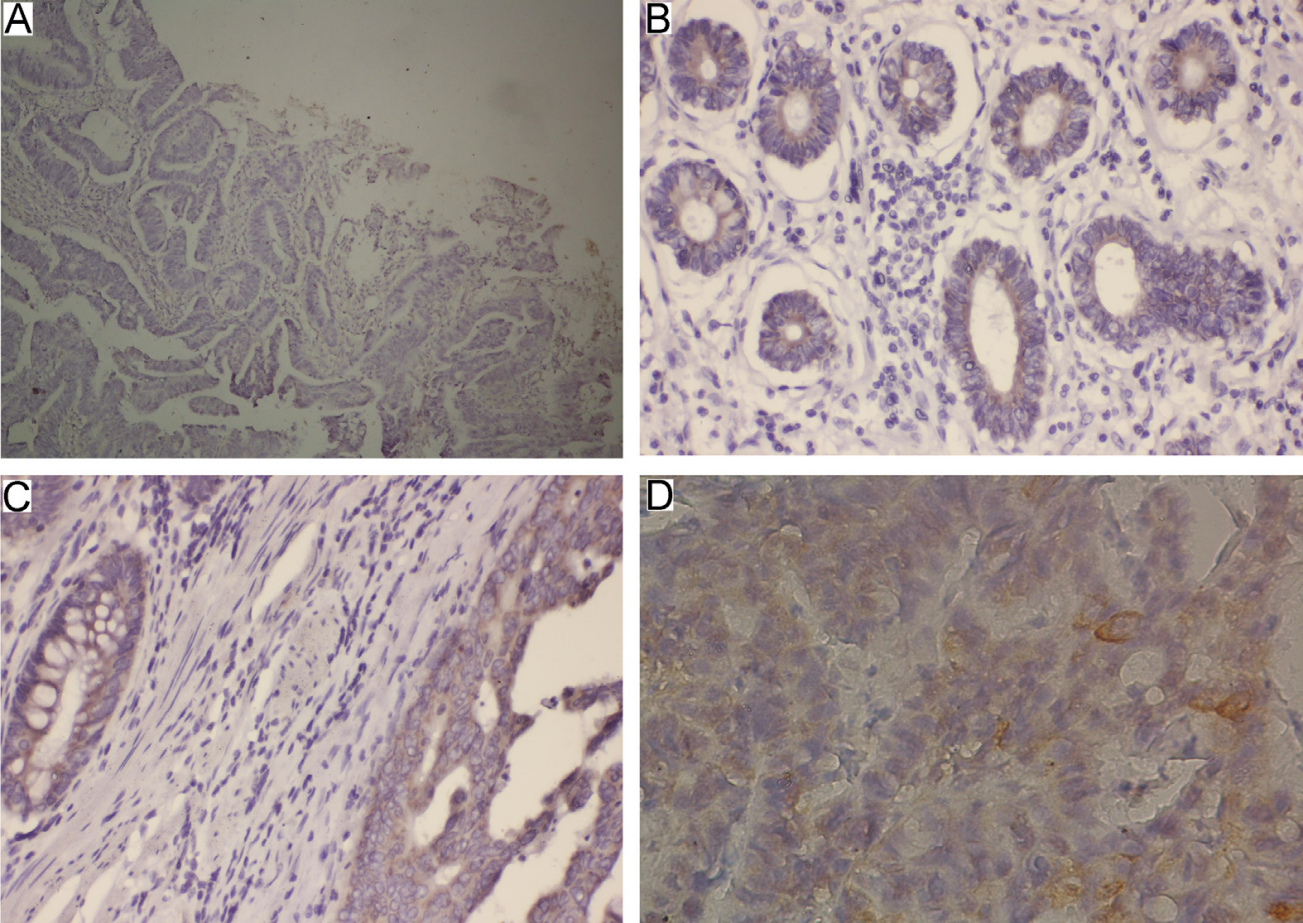
Representative images of immunohistochemical staining levels of FASN in gastric cancer tissues. (A) absent [0], (B) weak [1], (C) moderate [2], and (D) strong [3].

We used iTRAQ labeling combined with LC-ESI-MS/MS analysis to identify proteins that were differentially expressed in gastric cancer tissues compared with adjacent normal tissues. The analysis identified 431 proteins that were differentially expressed, of which 224 and 207 proteins were expressed at increased or decreased levels, respectively, in gastric cancer tissues (≥1.2-fold change) (Appendix A, Appendix A). Our data showed that the differential expressed proteins included the proteins to the cellular components, molecular functions, and biological process in gastric cancer tissues. The validation studies showed that FABP1 was highly expressed in GC tissues (Fig. 1). FASN was found to be overexpressed in gastric cancer tissues (Fig. 2 and Table 3). An in-depth analysis of the data is presented in the associated research article [1].

## 2 Experimental design, materials and methods

### 2.1 Materials

Paired gastric cancer and adjacent normal tissues (at least 5 cm from the cancer tissues) were selected from the tissue bank at the Department of Pathology of Affiliated Traditional Medicine Hospital of the Southwest Medical University. Any radiation therapy or chemotherapy was received by the patients before surgery. The clinical data including patient age, sex, diagnosis, and treatment strategies were collected. The iTRAQ reagents were purchased from Applied Biosystems (Foster City, CA, USA). The Polyclonal antibodies against fatty acid binding protein 1 (FABP1), fatty acid synthase (FASN), and β-actin were purchased from Cell Signaling Technology (Boston, MA, USA).

### 2.2 Methods

The experimental design and methods was as follows. First, we performed iTRAQ labeling and LC-ESI-MS/MS to analyze a test group of four paired samples of gastric cancer and adjacent normal tissues to identify differentially expressed proteins. For iTRAQ labeling, the first pair of gastric cancer and adjacent normal tissues in the test group was labeled with iTRAQ reagents 113 and 114, respectively, and the second, third, and fourth tissue pairs were labeled with iTRAQ reagents 115 and 116, 117 and 118, and 119 and 121, respectively.

The International Protein Index human sequence database and Mascot software Version 2.2 (Matrix Science, London, U.K.) were used to identify and quantify the peptide.

The Blast2 GO software (http://www.blast2go.com) and the Kyoto Encyclopedia of Genes and Genomes (KEGG) database were used to perform the Gene Ontology (GO) analysis and to identify the differential expressed proteins with metabolic pathways in gastric cancer tissues. Second, we performed IHC analysis of a validation group of 70 paired of samples of gastric cancer and adjacent normal tissues to confirm the differential expression of selected proteins (FABP1 and FASN) identified in the iTRAQ analysis of the test group.

## Appendix A Supplementary material

**Table 1.** Proteins identified as upregulated in gastric cancer tissues compared with adjacent normal tissues by iTRAQ-labeled MS/MS analysis. A 1.2-fold change was defined as the threshold for a significant expression change.

.

**Table 2.** Proteins identified as downregulated in gastric cancer tissues compared with adjacent normal tissues by iTRAQ-labeled MS/MS analysis. A 1.2-fold change was defined as the threshold for a significant expression change.

.

**Table 3.** Detailed list of more proteins identified as upregulated in gastric cancer tissues compared with adjacent normal tissues by iTRAQ-labeled MS/MS analysis.
